# The effect of organisational system on self-rated depression in a panel of male municipal firefighters

**DOI:** 10.1186/s40557-014-0044-x

**Published:** 2015-01-14

**Authors:** Se-Jin An, Yun Kyung Chung, Bong Hyun Kim, Kyeong Min Kwak, Jun-Seok Son, Jung-wan Koo, Young-Su Ju, Young-Jun Kwon

**Affiliations:** Department of occupational and environmental medicine, Hallym university sacred heart hospital, Anyang City, Republic of Korea; Department of occupational and environmental medicine, Changwon Samsung Hospital, Sungkyunkwan College of Medicine, Changwon City, Republic of Korea; Department of occupational and environmental medicine, Seoul St. Mary hospital, Catholic university of Medicine, Seoul City, Republic of Korea

**Keywords:** Depression, Organisational system, Municipality, Firefighter, Occupational stress

## Abstract

**Objectives:**

The present study evaluated the effects of job stress, including organisational system to self-rated depression through a panel study of male municipal firefighters in the Republic of Korea.

**Methods:**

A panel of 186 municipal firefighters reported self-rated depressive symptoms according to the Beck Depression Inventory (BDI). The effects of job stress were evaluated using the Korea Occupational Stress Scale, taken one year earlier and classified by the median value. Panel members were classified into Depression or Control groups according to BDI scores, with a cut-off level of ‘over mild depression’ in a follow-up survey.

**Results:**

The Depression group included 17 (9.1%) workers. Firefighters who scored high on occupational system had an 8.3 times greater risk of being assigned to the Depression group than those who had not (adjusted odds ratio [OR] = 8.03, 95% confidence interval (CI) = [1.73–37.22]). In contrast, job stress from a ‘difficult physical environment’ revealed negative risks related to being classified in the Depression group (AOR = 0.20, 95% CI = [0.04–0.92]).

**Conclusions:**

Although the healthy worker effect may be involved, job stress based on perceptions of organisational system was a strong risk factor for depression. A comprehensive approach should be considered that encompasses social issues when assessing or mental health in high-risk groups, as well as the practical issue of physiochemical hazards.

## Introduction

Firefighters perform a dangerous job that is an object of occupational medicine: they have long working hours [1] and are exposed to poor physiochemical surroundings. Other than risks during actual firefighting, job stress and adverse psychological health effects are also present [2]. In fact, epidemiological research has reported that firefighters display increased depression, post-traumatic stress disorder (PTSD), and lower quality of life that lead to mental pathology than do members of the general population [3]. In particular, research on adverse psychological effects found in fire officers has mostly focused on PTSD, but depression in general is the most frequently occurring mental disorder; research on the mental status of firefighters revealed a comorbidity of PTSD and depression, overlapping by as much as 16% [4]. Work stress or post-traumatic stress disorder resulting from having to meet the many demands of citizens can lead to extreme consequences such as suicide among firefighters, which attracts social attention. Besides physiochemical threats, other related elements reported to cause adverse psychological effects include conflicts in organisational system. However, there were a few limitations in previous studies resulting from the weaknesses inherent in cross-sectional studies, namely the lack of explanation of the causal relationship between exposed risks and health outcomes, and the inability to compare the influence of various subtypes of job stress such as organisational system and physical environment on depression. Therefore, this study aimed to examine the influence of job stress experienced one year ago to determine its effect on depression using a panel study design, a type of longitudinal study that can be used to analyse causal relationships.

## Material and methods

### Subjects

Two hundred and sixty firefighters were selected from 22 municipal fire stations including 5 police stands, 5 special rescue teams, and 5 inspection divisions in Seoul. The baseline survey was administered to 248 municipal firefighters. The 12 firefighters excluded from the baseline survey were 7 women, 2 patients under medical or psychiatric care, and 3 firefighter conscripts. After 12 months, the follow-up survey was administered to 210 firefighters; 38 did not respond to the follow-up survey due to transfer or migration. Twenty-four firefighters could not respond to the depression scale; therefore, 186 firefighters remained as the final study group. Informed consent regarding confidentiality for all subjects before enrolment.

### Methods

Through the initial and follow-up surveys, interviewer-assisted self-report questionnaires addressing socioeconomic and job-related characteristics, self-rated depression, and job stress were administered. The initial survey measured self-rated depression and job stress at 2005. The follow-up survey retested self-rated depression at 2006. Questionnaires were assigned to 248 subjects in total 2 yrs; 210 subjects responded to both the initial and follow-up surveys (response rate: 84.7%). Trained interviewers reviewed the questionnaires and checked for non-response. Socioeconomic characteristics collected were age, education, salary, and marital status. Job-related characteristics included working rank, type of occupation, shift work, and duration of work. Working rank was classified as one of 5 independent ranks according to the number of subordinates: firefighter, senior fire sergeant, fire sergeant, fire lieutenant, and above fire captain. Type of occupation included administrative, firefighting, rescue, and emergency. The type of occupational is fixed in principle. The type of shift was classified as no shift, 3 types of shift per day, 2 types of shift per day, and 1 shift of 24 h per day.

**Figure 1:**
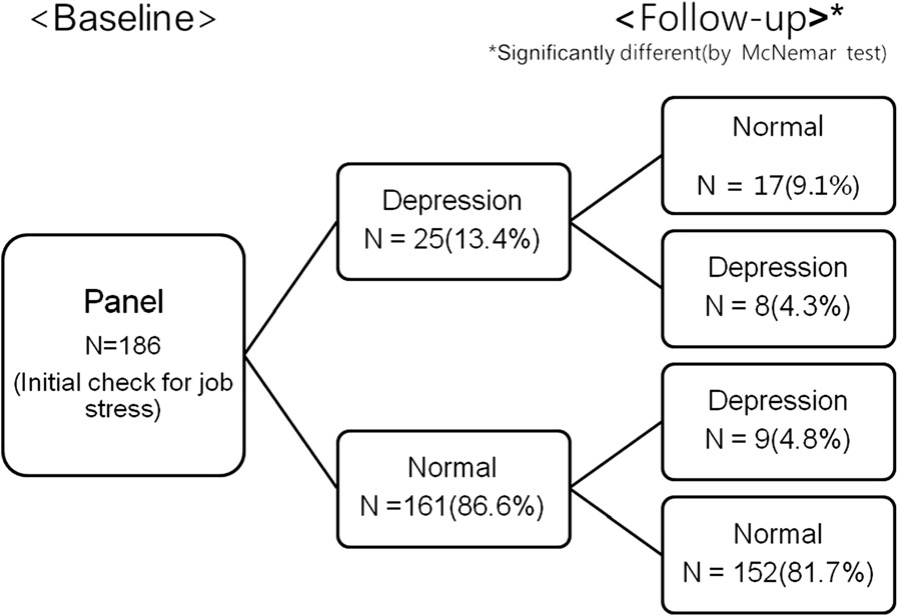
**The flowchart of study design and assignment of subjects.**

Self-rated depression was measured using the validated Korean version of the Beck Depression Inventory (BDI) at both the initial and follow-up surveys [5]. The Center for Epidemiologic Studies Depression Scale (CES-D) was completed during the initial survey. According to Beck’s criteria [6], a BDI under 10 was classified as ‘normal’. Through both the initial and follow-up surveys, the assignment to the 4 groups was performed as follows (Figure 1): normal-continuation (n = 152), depression to normal (n = 17), normal to depression (n = 9), and depression-continuation (n = 8). The operational definition established that the Depression group (n = 17) included the former 2 groups and the Control group (n = 169) included the latter 2 groups.

The self-report questionnaire for occupational stress was administered during the initial study. The short form of the Korea Occupational Stress Scale (KOSS) is composed of the following eight subscales: difficult physical environment, high job demand, insufficient job control, inadequate social support, job insecurity, organisational system, lack of reward, and discomfort in the occupational climate [7].

Organisational system was measured with 7 statements, as follows: ‘The organisational policy of my company is fair and reasonable’ ,. ‘My company provides me with sufficient organisational support’ ,. ‘Departments cooperate with each other without conflict’ ,. ‘All employees cooperate in harmony for the company’ ,. ‘I have opportunities and channels to talk about my ideas’ ,. ‘I expect my career development and promotion to progress as I have planned’ ,. and ‘My current status is appropriate for my education and career’.

The median score of the study subjects on each subscale was used as the cut-off to classify the high and low job stress groups.

Classification as ‘normal’ or ‘not significantly changed’ after 12 months was analysed by McNemar’s test. Between the Depression and Control groups, socioeconomic and job-related characteristics were comparatively analysed with chi-square and t-tests. Crude and adjusted odds ratios using covariates of age, rank, and shift work were calculated with 95% confidence intervals using multiple logistic regression.

## Results

The point prevalence of self-rated depression was 13.4% at the initial survey and 9.1% at the follow-up survey. The difference of classification into ‘normal’ or ‘self-rated depression’ was significantly different through the 12 months of follow-up (p < 0.05, McNemar’s test) (Figure 1). The socioeconomic characteristics of subjects in the Depression group were compared with the Control group. The majority of study subjects were married (87.6%) high school graduates (55%) in their 30s (41.9%) or 40s (42.5%), with monthly salaries starting at 2,000 US Dollar (40.8%). No statistically significant differences were shown between the Depression group and the Control group. Additionally, none of the job-related characteristics, including working class, job content, and shift work presented significant differences (Table 1).

**Table 1:** **General characteristics of subjects**

**General characteristics**		**Total**		**Depression**		**Control**		**P-value** ^*^
		**(N = 186)**		**(N = 17)**		**(N = 169)**		
Age								0.287
	29	3	(1.7)^†^	0	0	4	2.4	
	30-39	78	41.9	4	23.5	74	43.8	
	40-49	79	42.5	11	64.7	68	40.2	
	50-	25	13.4	2	11.8	23	13.6	
	Subtotal	186	100	17	100	169	100	
Education								0.740
	-Middle school	4	2.2	0	0	4	2.5	
	High school	99	55	9	52.9	90	55.2	
	2-year university	36	20	5	29.4	31	19	
	4 –years college-	41	22.8	3	17.6	38	23.3	
	Subtotal	180	100	17	100	163	100	
Salary in a month								0.418
	<$2000	9	5	0	0	9	5.5	
	$2000-2999	73	40.8	7	43.8	66	40.5	
	$3000-3999	69	38.5	5	31.3	64	39.3	
	$4000-	28	15.6	4	25	24	14.7	
	Subtotal	179	100	16	100	163	100	
Marriage								0.815
	Married	162	87.6	15	88.2	147	87.5	
	Single	17	9.2	2	11.8	15	8.9	
	Divorced or seperated	6	3.2	0	0	6	3.6	
	Subtotal	185	100	17	100	168	100	
Working class								0.268
	Firefighter	26	(14.1)^†^	2	11.8	24	14.3	
	Vice director	85	45.9	5	29.4	80	47.6	
	Director	59	31.9	8	47.1	51	30.4	
	Chief director	6	3.2	0	0	6	3.6	
	Above president	9	4.9	2	11.8	7	4.2	
	Subtotal	185	100	17	100	168	100	
Job content								0.721
	Administrative	49	26.8	5	29.4	44	26.5	
	Firefighting	81	44.3	6	35.3	75	45.2	
	Rescue (accident)	31	16.9	3	17.6	28	16.9	
	Emergency (health)	22	12	3	17.6	19	11.4	
	Subtotal	183	100	17	100	166	100	
Shiftwork								0.545
	None	38	20.7	2	13.3	36	21.3	
	3 shift	6	3.3	1	6.7	5	3	
	2 shift	44	23.9	5	33.3	39	23.1	
	1 shift for 24 hrs	96	52.2	7	46.7	89	52.7	
	Subtotal	184	100	15	100	169	100	

*Chi-square test (P < 0.05).

^†^N(%).

The involvement of job stress in classification into the Depression group was analysed through logistic regression (Table 2). The risk of being classified in the Depression group was 8 times higher for those in the high occupational system stress group than for those in the low stress group regardless of subjects’ age, rank, and type of shift work (COR = 8.34, 95% CI [1.82–38.21]; AOR = 8.03, 95% CI [1.73–37.22]). In contrast, subjects in the high difficult physical environment stress group showed a 0.2 times lower risk of being classified in the Depression group (COR = 0.19, 95% CI [0.04–0.86]; AOR = 0.20, 95% CI [0.04–0.92]).

**Table 2:** **The logistic regression of korean occupational job stress scales on self-rated depression**

**Sub-items in job stress** ^**†**^		**Crude**	**95% CI**		**Adjusted**	**95% CI**	
		**Odd ratio**			**Odd ratio** *		
Total score							
	Low group	1.00			1.00		
	High group	2.44	0.80	−7.48	2.50	0.80	−7.78
Physical environment							
	Low group	1.00			1.00		
	High group	0.19	0.04	−0.86	0.20	0.04	−0.92
Job demand							
	Low group	1.00			1.00		
	High group	2.19	0.77	−6.21	2.24	0.78	−6.41
Job control							
	Low group	1.00			1.00		
	High group	0.97	0.33	−2.80	0.92	0.32	−2.70
Lack of social support							
	Low group	1.00			1.00		
	High group	0.86	0.30	−2.44	0.80	0.28	−2.32
Job instability							
	Low group	1.00			1.00		
	High group	1.14	0.23	−5.77	1.14	0.23	−5.77
Organisational system							
	Low group	1.00			1.00		
	High group	8.34	1.82	−38.21	8.03	1.73	−37.22
Lack of reward							
	Low group	1.00			1.00		
	High group	1.58	0.56	−4.46	1.58	0.55	−4.49
Organizational climate							
	Low group	1.00			1.00		
	High group	0.75	0.27	−2.14	0.65	0.22	−1.91

*Odd ratio was adjusted by age, job class, and shiftwork.

^**†**^High group was divided by median score of each sub-items of job stress.

## Discussion

The results of the study indicate that male firefighters who organisational system were 8 times more likely to become depressed than those who did not. This result was consistently observed even after adjusting for age and job position. This is similar to other previous studies reporting a relationship between organisational justice and depression. A cohort study conducted with civil servants in England showed that adverse change system increased psychiatric risks [8], as well as the risk of poor self-rated health [9]. In addition, previous studies were conducted to examine the connection between job stress and depression in firefighters. As a result, a subscale of job stress related to role conflict (OR = 1.8) and lower self-esteem (OR = 5.8) was presented as a risk factor. In particular, firefighters working for 24 hours showed work stress factors similar to procedural injustice such as inter-group conflict (OR = 1.7) and role ambiguity (OR = 1.6) as risk factors for depression.

This study, however, found that the difficult physical environment of firefighters had a negative association on depression (OR = 0.20, 95% CI [0.04–0.92]). The public workers such as rescue workers [10] and police officers [11] are reported that they are relatively resilient to mental health problems as they have been trained to cope with critical incidents since the beginning of their employment. Notably, this can also be seen as a result of the healthy worker effect, which can introduce a bias. Firefighters are selectively placed because they are physically and mentally healthier than the general population. Moreover, it is generally known that firefighters experience extremely strong social bonding, referred to as a ‘brotherhood’; this shows that the basic organisational culture of firefighters is likely to attenuate the influence of job stress on depression [12].

On the other hands, it has been reported that subjectively perceived incidents are classically more influential than objective incidents or situations as the pathologic mechanism of depression [13]; a lack of coping skills for responding to this is known to be an important mechanism [14]. The results of this study, that organisational system felt by individuals acts as a stronger risk factor for depression than frequently experienced job stress caused by a difficult physical environment, contrast with the results of existing studies on the pathogenesis of depression.

However, while the mental prescription for physical incidents can be filled by superficial contemplation, an invisible injustice at organisational system is a problem that is difficult to actively address and resolve.

A qualitative study in Korea indicated that in organisational systems, firefighters’ complaints include low allowances, bottlenecked promotions, and a rigid corporate culture [2]. Using the same job stress questionnaire used in this study, research conducted by KOSS found that organisational system, as a job stress factor of firefighters, was significantly higher than that found in the general population [15].

KOSS was validated in Korea and then applied to various types of occupations, presenting reference values for Korean workers [7] and a discussion to understand items through correlation analysis among the subscales. And organisational system in KOSS is also referred to as organisational culture, showing a lack of reward and strong correlation (r = 0.67) with other KOSS subscales [16]. A previous study [17] noted effect-reward imbalance as a predictor of poor mental health. Moreover, the ‘lack of reward’ could have detrimental effects on self-esteem. A Japanese study similar to this one investigated risk factors of depression in firefighters and reported that low self-esteem was five times more relevant to depression among them. Meanwhile, relational injustice is a component of organisational injustice that most previous studies have dealt with [18,19]. Studies on the absence of sickness [20] or self-rated health [9] have focused mostly on relational injustice. This study differs from the existing research in that it focused on procedural injustice. In the Korean municipal worker environment, organisational system is realistically close to procedural injustice. In fact, the internal congestion of promotions for municipal workers is attracting attention from society. A survey [21] showing that promotion was influenced by personal connections internally supports the assumption that unfairness is widespread in the decision-making process of the municipal employment hierarchy. This study found that such a situation might act as a severe stressor for staff in the organisation, and, if chronic, could cause depression after a year. In contrast, research that examined the relevance to depression using the same KOSS survey of all workers in Korea unexpectedly showed that organisational system did not have a great influence on depression [16,18,22]. However, this study discovered the adverse effects of organisational system on mental health, similar to previous research findings on firefighters, instead of the above study targeting all workers in Korea. In particular, unlike the aforementioned studies, the strength of this study lies in its longitudinal design and clear temporal relationships, as well as in the maintenance of internal consistency by restricting subjects to a certain panel of firefighters.

Injustice at organisational system also differs according to type of health effects. Stansfeld reported that organisational injustice is a major factor in short-term absence due to illness in women and long-term absence in men [23].

In our study, female firefighters were excluded from the final study group. The duties of female firefighters were mostly administrative jobs, different from those of male firefighters. Furthermore, subjects who presented as depressed in the initial survey were excluded to avoid confounding results caused by medication, and because this could be an interruption of the natural course of the study.

There are several known risk factors for depression including young age, being unmarried, and low socioeconomic status [24]. In our results, a univariate analysis of the above known risk factors according to depression revealed that they did not show any statistically significant association with depression. The point prevalence of depression in our study was 13.4% at the initial survey and 9.1% at the follow up survey. In the general population of Korea, the point prevalence of depression is 5%, 2.5% for lifetime prevalence according to an interview-based survey [25]. Using CES-D, the same self-rating scale used in the present study, the point prevalence for males in the general population was found to be 6.5% [26]. A similar study of depression in firefighters in Taiwan found a point prevalence for depression of 5.4%, and 10.5% for PTSD. In the same study, current PTSD status was shown to be a significant predictor for current major depression (OR = 1.157) [27]. Among firefighters present at the World Trade Center incident (also called 9/11), the point prevalence of combined PTSD and depression was 16.1%, which is higher than the prevalence of depression alone (5.9%). Because of limitations in the self-rated scale, the prevalence of depression in our results could not be adequately discriminated from depression co-morbid with other mental disorders. However, the healthy worker effect likely attenuated symptoms, so the actual prevalence could be understated due to the effect of bias toward the null. Besides, the size of depression group is slightly small and might be limitation of our study design. Because of panel study, the size and spectrum of participant was small and narrow and the depression group as well.

Notabley, this study was designed as a longitudinal panel study design which have a strength that it could be catch-up the level and trend of change of variance in single panel at the dynamic view point among other longitudinal studies. The dynamic changes of variance and status should be plausible evidences of the policy and management in a homogeneous panel. In case of this study, there is additional study about the depression-recovered group who would be also important group in practical view point of job-fitness. The risk factor and natural course of mild self-rated depression of firefighter should be noticed for management of public health. On the other hands, panel study design has a limitation to show exact causal relationship than the other longitudinal study design. A longitudinal study with incident case at the follow-up survey would be most appropriate to evaluated the causal relationships rather than cross-sectional study.

In terms of keep validity of panel study, the attrition rate should be worried for overestimation of variance of retention group. In our study, the follow-up loss due to job circulation was 38 subjects (13%) out of the final panel. Subgroup analysis of the 38 lost follow-up participants and high group in physical environment was conducted to assess the internal consistency of the panel. Because the distribution of socioeconomic status was not different statistically from that of the final subjects, the original characteristics of the panel were maintained.

Furthermore, panel studies are vulnerable to the weaknesses of instruments, which could be memorized by the subjects in the panel and therefore present biased results in the final analysis. Therefore, the questionnaires were varied to prevent subjects from learning the pattern of answers. For instance, the 2 types of self-rated scales for depression (the BDI and CES-D) were administered but only the BDI was used at the time of the follow-up survey. However, the validity of the self-rated scales of the BDI was used to measure symptoms of depression, not clinical diagnosis. Comparing BDI scales was suggested to be valid, and there should be little discordance between the questionnaire and actual clinical diagnosis [28]. A survey of 120 psychiatrists found that 70.89% of them suggest the BDI, with their suggestion confirmed by clinical practices [29].

## Conclusions

Despite several limitations, this study has attempted to identify a effect of job stress to self-rated depression in municipal firefighters through a longitudinal panel study design. Based on our results, it is reasonable to propose that the development of male municipal firefighters should focus on not only job stress resulting from a difficult physical environment, but also organisational system. A comprehensive approach should be considered that incorporates social and academic issues in assessments of psychosocial risk or mental health in high-risk groups in addition to the practical issue of physiochemical hazards.

## References

[1] Lee D (2004). An Empirical Study on the Relations of job Stressor and Morale of the Police Officer.

[2] Guidotti TL (1992). Human factors in firefighting: ergonomic-, cardiopulmonary-. and psychogenic stress-related issures. Int Arch Pccup Environ Health.

[3] Kang BS (2006). Work Related Disease and Health Management of Fire Officer.

[4] Chiu S, Niles JK, Webber MP, Zeig-Owens R, Gustave J, Lee R, Rizzotto L, Kelly KJ, Cohen HW, Prezant DJ (2011). Evaluating risk factors and possible mediation effects in posttraumatic depression and posttraumatic stress disorder comorbidity. Public Health Rep.

[5] Hahn HM, Yum TH, Shin YW, Kim KH, Yoon DJ, Chung KJ (1986). A standardization study of beck depression inventory in Korea. J Korean Neuropsychiatr Ass.

[6] Beck AT, Ward CH, Mendelson M, Mock J, Erbaugh J (1961). An inventory for measuring depression. Arch Gen Psychiatry.

[7] Chang S, Koh S, Kang D, Kim S, Kang M, Lee C, Chung J, Cho J, Son M, Chae C, Kim J, Kim J, Kim H, Roh S, Park J, Woo J, Kim S, Kim J, Ha M, Park J, Rhee K, Kim H, Kong J, Kim I, Kim J, Park J, Huyun S, Son D (2005). Developing an occupational stress scale for Korean employees. Korean J Occup Environ Med.

[8] Ferrie JE, Head J, Shipley MJ, Vahtera J, Marmot MG, Kivimaki M (2006). Injustice at work and incidence of psychiatric morbidity: the Whitehall II study. Occup Environ Med.

[9] Kivimaki M, Ferrie JE, Head J, Shipley MJ, Vahtera J, Marmot MG (2004). Organisational justice and change in justice as predictors of employee health: the Whitehall II study. J Epidemiol Community Health.

[10] Van der Velden PG, Rademaker AR, Vermetten E, Portengen MA, Yzermans JC, Grievink L: **Police officers: a high-risk group for the development of mental health disturbances? A cohort study.** *BMJ* 2013. 10.1136/bmjopen-2012-001720. 10.1136/bmjopen-2012-001720 PMC3563131 23355659

[11] Lalic H, Bukmir L, Ferhatovic M (2007). Examining psychic consequences in firefighters exposed to stress. Coll Antropol.

[12] Carey MG, Al-Zaiti SS, Dean GE, Sessanna L, Finnell DS (2011). Sleep problems, depression, substance use, social bonding, and quality of life in professional firefighters. J Occup Environ Med.

[13] Tennant C (2002). Life events, stress and depression: a review of recent findings. Aust N Z J Psychiatry.

[14] Sargeant JK, Bruce ML, Florio LP, Weissman MM (1990). Factors associated with 1-year outcome of major depression in the community. Arch Gen Psychiatry.

[15] Kim TW, Kim KS, Ahn YS (2010). Relationship between job stress and depressive symptoms among field firefighters. Korean J Occup Environ Med.

[16] Cho JJ, Kim JY, Chang SJ, Fiedler N, Koh SB, Crabtree BF, Kang DM, Kim YK, Choi YH (2010). Occupational stress and depression in Korean employees. Int Arch Occup Environ Health.

[17] Kivimaki M, Vahtera J, Elovainio M, Virtanen M, Siegrist J (2007). Effort-reward imbalance, procedural injustice and relational injustice as psychosocial predictors of health: complementary or redundant models?. Occup Environ Med.

[18] Colquitt JA, Conlon DE, Wesson MJ, Porter CO, Ng KY (2001). Justice at the millennium: a meta-analytic review of 25 years of organizational justice research. J Appl Psychol.

[19] Elovainio M, Kivimaki M, Vahtera J (2002). Organizational justice: evidence of a new psychosocial predictor of health. Am J Public Health.

[20] Elovainio M, Van den Bos K, Linna A, Kivimaki M, Ala-Mursula L, Pentti J, Vahtera J (2005). Combined effects of uncertainty and organizational justice on employee health: testing the uncertainty management model of fairness judgments among Finnish public sector employees. Soc Sci Med.

[21] Lee M, Jeong PH (2006). With emphasis on the perceptions of local public officials; an exploratory study on the misuse of personnel management power by local governors. Korean corrupation studies review.

[22] Park SG, Min KB, Chang SJ, Kim HC, Min JY (2009). Job stress and depressive symptoms among Korean employees: the effects of culture on work. Int Arch Occup Environ Health.

[23] Stansfeld SA, Fuhrer R, Head J, Ferrie J, Shipley M (1997). Work and psychiatric disorder in the Whitehall II study. J Psychosom Res.

[24] Hardeveld F, Spijker J, De Graaf R, Nolen WA, Beekman AT (2010). Prevalence and predictors of recurrence of major depressive disorder in the adult population. Acta Psychiatr Scand.

[25] Ministry of Health and Welfare (2006). The Epidemiological Survey of Psychiatric Illnesses in Korea.

[26] Cho MJ, Nam JJ, Suh GH (1998). Prevalence of symptoms of depression in a nationwide sample of Korean adults. Psychiatry Res.

[27] Chen YS, Chen MC, Chou FH, Sun FC, Chen PC, Tsai KY, Chao SS (2007). The relationship between quality of life and posttraumatic stress disorder or major depression for firefighters in Kaohsiung, Taiwan. Qual Life Res.

[28] Lee YH, Song JY (1991). A Study of the reliability and the validity of the BDI, SDS, and MMPI-D scales. Korean J Clin Psychol.

[29] Lee EJ, Kim JB, Shin IH, Lim KH, Lee SH, Cho GA, Sung HM, Jung SW, Zmimmerman M, Lee Y (2010). Current use of depression rating scales in mental health setting. Psychiatry Investig.

